# Assessing the psychometric properties of the Arabic version of the Nursing Practice Readiness Scale among Saudi nursing students

**DOI:** 10.1371/journal.pone.0289088

**Published:** 2023-07-27

**Authors:** Nahed Alquwez, Jonas Preposi Cruz, Ejercito Balay-odao

**Affiliations:** 1 Department of Nursing, College of Applied Medical Sciences, Shaqra University, Dawadmi, Saudi Arabia; 2 Department of Medicine, School of Medicine, Nazarbayev University, Kerey and Zhanibek, Astana, Kazakhstan; University of Copenhagen: Kobenhavns Universitet, DENMARK

## Abstract

Readiness to practice nursing is essential for nursing students to ensure that they are conscientious, have a sense of responsibility, and are rational in performing their clinical practice. This study tested the psychometric properties of the “Nursing Practice Readiness Scale” Arabic version (NPRS-A) to assess Saudi nursing students’ readiness to practice nursing in clinical settings. This study used a methodological design to examine the psychometric properties of the NPRS-A among 373 nursing students in Saudi Arabia. The findings provide evidence of the NPRS-A’s good content validity. The PCA revealed five distinct components with a "cumulative variance explained" of 69.2%. The test of difference on the nursing practice preparedness between students from different levels showed that students in the 2^nd^ year were less prepared than junior and senior nursing students. The correlation test showed that a higher GPA and higher self-reported readiness are more ready to practice nursing in clinical settings. The research showed an overall Cronbach’s alpha of 0.957. The establishment of the NPRS-A is significant, not only in Saudi Arabia but other Arabic-speaking countries. Nurse educators and nursing education policymakers can use this version to assess nursing students’ learning needs to be prepared to practice nursing.

## Introduction

Since the beginning of nursing education, knowledge, skill, and attitudes have been developed to prepare the student nurse to deliver safe and quality patient care. Shaping nursing students’ knowledge, skills, and attitudes will help them transition from academic education to professional practice. According to Aldosari et al. [1], nurses usually experience poor adaptation to their work demands and anxiety during the transition. Opoku et al. [2] further mentioned that many new graduate nurses struggle during the transition period; most of them experience the inability to adapt to multitasking and have a good working and interpersonal relationships with their colleagues. Clinical practice is essential to understanding nursing knowledge, skills, and attitude as a scaffold for student nurses in applying moral and ethical reasoning and clinical judgment. Clinical practice readiness is the hallmark of nurse competence. In this modern world, the clinical setting is increasingly challenging due to new health problems and the advancement of technology, which requires developed clinical practice [3]. Thus, understanding nursing student readiness for clinical practice is essential. In Saudi Arabia, a study on nursing students’ readiness for clinical practice received less attention from the researchers. One of the reasons for this could be the need for a valid and reliable instrument to assess this construct. Therefore, there is a need for a valid scale within a country’s context. The “Nursing Practice Readiness Scale” (NPRS) is a new scale that can be employed to assess nursing students’ readiness to practice nursing in the clinical area [4]. Thus, this tool was adapted to the Arabic language (NPRS-A) and was tested for validity and reliability among Saudi students.

### Background of the study

Nursing education’s primary responsibility is to develop nursing students’ knowledge, attitudes, and competence to practice nursing in the real world. Readiness to practice nursing is essential for nursing students to ensure that they are conscientious, have a sense of responsibility, and are rational in performing their clinical practice [5]. Being ready to practice nursing means that a nursing student develops the ability to respect human dignity and rights, be compassionate, and provide competent nursing care without bias [6]. It also infers the ability of the nursing student to be mindful and take a stance against unlawful nursing care during training while working in a team and clinical practice [5]. Although nursing students recognize the importance of being ready to practice nursing, some students may need to recognize the importance of professional nursing practice values and attributes in their future work, which could impact their preparedness to practice nursing.

The nursing undergraduate course’s objective is to develop nursing students’ knowledge, skills, and attitude to apply in nursing practice, education, and research. However, studies prove that newly graduated nurses need to gain the skill and be more competent in nursing care [7, 8]. The lack of student preparation is connected with the mediocre learning method during nursing programs [9]. It was also noted that the education system influences student nurses’ lack of practice readiness. The consistency of theory and practice and the need for updated nursing lecture content affect student practice readiness [8]. “Theory and practice gap” is one of the factors in the nurses’ shock experienced during the transition period [10].

The competence of nursing students is a product of their learning process. Lack of preparedness to practice nursing is due to inadequate knowledge and clinical practice competence, which could lead to the careless provision of poor-quality care and patient safety risks. Furthermore, Park et al. [11] mentioned that nursing students need to be more professional during their clinical exposure when they report or falsify their patients’ data, such as reporting not assessing and unmeasured vital signs. Professional development of nursing students’ professional values is essential to comply with clinical practice standards. Future nurses must profess a high professional value to practice proper and ethical conduct in providing clinical practice [12]. Thus, nursing study plays a special part in the professional values improvement of student nurses to prepare them to practice nursing.

The importance of the preparation of nursing students to practice nursing is underscored. Knowing that nurses hold the highest number in the health care system workforce, their clinical practice readiness is essential to understand. However, on a closer look at the literature search, there are no apparent reasons for the factors affecting student nurses’ practice readiness. There is also a need for more literature assessing this construct in various parts of the world, including Saudi Arabia. This could be due to the unavailable tool that can be used to measure the preparedness of nursing students to practice professionally. The NPRS is one of the few tools that had been established to measure this construct [4]. This tool was developed using a rigorous method, including framework development, literature review, and qualitative interviews. The tool has undergone stringent validity and reliability tests to support its good psychometric properties in measuring the new graduate nurse’s preparedness to practice nursing [4]. Therefore, this study sought to adapt the tool to the Arabic language and for nursing students to assess their preparedness to practice nursing in Saudi Arabia and other Arab-speaking countries.

### Aims

This study tested the NPRS-A’s psychometric properties when assessing Saudi nursing students’ readiness to practice nursing in clinical settings.

## Materials and methods

This research is a methodological study. The reporting of this study followed the “STROBE checklist for cross-sectional studies” (S1 Checklist). This study was conducted at Shaqra University in Saudi Arabia. The inclusion criteria to be eligible to participate consisted of: (1) Saudi nationality, (2) being enrolled in the BSN program in the university, and (3) consenting to participate. The sample size was determined based on 10 samples for every item on the scale ratio [13]. Since there were 35 items on the scale, the estimated sample size was 350 Saudi nursing students. Using the convenience sampling method, 373 Saudi nursing students were surveyed.

### Instrument

A pen-and-paper survey was employed to gather data for the validity and reliability of the NPRS-A. The instrument comprised two sections. Section 1 contained questions to gather information about the respondent’s demographic characteristics, such as age, sex, BSN year level, latest GPA, and perceived readiness to practice nursing using a 1 to 10 rating scale. Part 2 was the NPRS-A. The NPRS-A was created following the guidelines for “cross-cultural adaptation of measurement scales” [14]. Accordingly, a forward-backward translation method was employed. According to Beaton et al. [13], five steps were observed in the translation process: “forward translation, synthesis, back-translation, expert committee review, and pretesting.” For the “forward translation,” two bilingual translators (one who knew the concept of the survey and one who was unaware about the survey) translated the study into Arabic. These two translations were done independently. Another bilingual translator integrated the two Arabic translations to form the Arabic version (“synthesis”). This version was translated back to English by two independent translators in the “back-translation” step. A panel reviewed the two back-translations and the Arabic versions to examine the equivalency of the Arabic and English versions. They also rated the NPRS-A’s content validity. Five clinical nurses with either Master’s in nursing or Doctorate in nursing evaluated the NPRS-A’s CV. The experts were also teaching in a nursing program at a university and were in-charge of the clinical experiences of the nursing students. After the panel approved the NPRS-A, 30 Saudi nursing students responded to the tool in “pretesting”. The students reported the clarity of the Arabic version, and thus no changes were made at this stage.

The “Nursing Practice Readiness Scale” (NPRS) is a recently developed scale that measures the readiness to practice nursing among newly graduated nurses [4]. The NPRS consists of 35 items measuring five areas/factors of “nursing practice readiness”: “clinical judgment and nursing performance, professional attitudes, patient-centeredness, self-regulation, and collaborative interpersonal relationships” [4]. The scale uses a 4-point Likert scale: 1 = “strongly disagree” to 4 = “strongly agree.” Scores can be calculated by getting the overall and subscale means, with higher means signifying more readiness to practice nursing. NPRS’s validity was supported by content and construct validities using “exploratory factor analysis, confirmatory factor analysis, and criterion-related.” The reliability was supported by Cronbach’s α computation (whole scale = 0.90, subscales = 0.83–0.85) [4].

### Ethical consideration

This study is part of a research project protocol that was reviewed and approved by the Ethics Review Committee of Shaqra University (ERC_SU_20220086). The study was conducted at the highest ethical standards, following the recommendations of the “Declaration of Helsinki” and the Ethics Research Committee. Participation was voluntary. The participants signed a written informed consent after receiving thorough information. In the informed consent process, researchers discussed with the potential participants what the study is all about, its objectives, significance, and processes. The potential risks and benefits of participation were also discussed with them. Voluntary participation was reiterated. The confidentiality and privacy of the respondents and the data were protected throughout the study. Only the researchers had access to the data. Responded surveys were put in a secured cabinet, and data were secured in a password-protected computer. No personal identifications were collected from the participants. The researchers did not have access to any information that can distinguish the individual respondents during or after data gathering. The authors of the original scale gave the researchers permission to use and translate the scale into the Arabic language.

### Data collection

Two researchers gathered the data during the break of the respondents. The study’s information was explained to them, and those who agreed to participate signed the informed consent. After this, the survey was handed to the students. They were given enough time to answer the survey. The data gathering was conducted for September 27 to October 31, 2022.

### Data analysis

The “item-level content validity” (I-CVI) and “scale-level content validity” (S-CVI/Ave) indices were calculated following the recommendations of Polit and Beck [15]. The experts evaluated the relevance of each item to the underlying construct “nursing practice readiness” using a four-point scale: 1 for “not relevant,” 2 for “somewhat relevant,” 3 for “quite relevant,” and 4 for “highly relevant.” This was the most frequently employed method of assessing content validity. The four-point scale also avoided neutral and ambivalent responses [15]. According to Polit and Beck [15], scores of 1 and 2 were coded as 0, and 3 and 4 were coded as 1. The acceptable I-CVI value and S-CVI/Ave were 1 [15]. The “Item-total correlations” (ITC) was estimated for internal construct validity. NPRS-A’s item mean, and standard deviation were estimated. A “Principal component analysis” (PCA) with “Varimax rotation” was performed on the NPRS-A to extract its components. For extracting the components, “Eigenvalue” of 1 and “factor loadings of >0.40 were used [16]. “Kaiser–Meyer–Olkin (KMO) test” and “Barlett’s test of sphericity” were analyzed. Hypothesis testing was also used to support the NPRS-A’s construct validity. The hypotheses were tested using Pearson’s correlation and a One-way Analysis of variance with a posthoc analysis Tukey HSD test. Internal consistency reliability was supported by Cronbach’s alpha calculation of the scale and its components. An alpha greater >0.70 is acceptable [16]. All analyses were done utilizing the SPSS version 22.0.

## Results

The respondents comprise mostly females (71.0%) with a mean age of 20.94 (SD = 1.42) years. About 48.8% of the students were in the 3^rd^ year level (48.8%), while 2^nd^ and 4^th^ year students constitute 29.5% and 21.7% of the respondents, respectively. The average the latest GPA of the students was 4.06 (SD = 0.48). The respondents perceived their readiness to practice nursing as moderate, with a mean score of 7.66 (SD = 2.51) on a scale of 1 to 10 (Table 1).

**Table 1:** Characteristics of the respondents (n = 373).

Variable	n	%
Gender		
Male	108	29.0
Female	265	71.0
Academic year level		
2nd year	110	29.5
3rd year	182	48.8
4th year	81	21.7
	Mean (SD)	Range
Age	20.94 (1.42)	18–27
Latest GPA	4.06 (0.48)	2.30–5.00
Perceived readiness to practice nursing	7.66 (2.51)	1.00–10.00

### Descriptive analysis of the scale items

As indicated in Table 2, the mean item scores ranged from 2.92 (SD = 0.87) for item “I have the nursing techniques practiced frequently in the ward” to 3.47 (SD = 0.67) for the item “I try to empathize with the patient’s situation,” indicating moderate to high levels of nursing practice readiness. The ITC values ranged from 0.30 to 0.77, implying that each item had an acceptable association with the whole scale. Thus, all items were eligible to be included in the PCA.

**Table 2:** Results of descriptive analyses on the scale items (n = 373).

No.	Item	Mean	SD	Item-to-total correlation
1	I have the nursing knowledge especially required in the ward.	2.95	0.81	0.64
2	I have the nursing techniques practiced frequently in the ward.	2.92	0.87	0.64
3	When nursing a patient, it is possible for me to determine when a request for help is needed for abnormal conditions.	3.28	0.84	0.70
4	I can understand the evidence for frequently performed nursing activities in the ward.	3.15	0.84	0.69
5	I have necessary knowledge of nursing majors.	3.06	0.88	0.62
6	I can determine the patient’s condition considering the nursing interventions implemented.	3.03	0.86	0.75
7	I can apply knowledge and skills from major and elective courses in an integrated manner.	3.17	0.84	0.73
8	I can use scientific evidence to identify changes in the patient’s condition.	3.08	0.87	0.69
9	I can assess the patient’s physical, psychological, and social condition.	3.15	0.88	0.72
10	I can use the electronic medical record system skillfully.	3.01	0.93	0.69
11	I can collect scientific information necessary for patient care from a variety of sources.	3.11	0.88	0.76
12	I know what nursing activities are needed according to workflow.	3.09	0.86	0.76
13	I can prioritize nursing activities according to the situation.	3.07	0.88	0.77
14	I can carry out all planned nursing activities within a given time.	3.05	0.85	0.76
15	I know the main duties in the ward.	3.17	0.85	0.71
16	I can provide patient care while implementing safety management guidelines.	3.10	0.84	0.67
17	I can cope with stress positively.	3.04	0.89	0.73
18	I can maintain a balance between work and life.	3.05	0.86	0.75
19	I think I’m a necessary member of the ward.	3.13	0.88	0.74
20	I can seek help in stressful situations from a coworker or preceptor.	3.16	0.92	0.72
21	I can stay calm in a psychologically stressful situation.	3.08	0.92	0.73
22	I can provide patient care independently without relying on peers.	3.08	0.91	0.73
23	I have faith in my own abilities and potential.	3.25	0.90	0.73
24	I can respond to patients and caregivers in a professional manner.	3.24	0.92	0.77
25	I can protect the privacy of the patient.	3.43	0.68	0.38
26	I can encourage the patient to express their feelings.	3.41	0.72	0.37
27	I try to empathize with the patient’s situation.	3.47	0.67	0.35
28	I can be actively engaged in work.	3.45	0.68	0.34
29	I treat patients and medical staff with respect.	3.44	0.71	0.35
30	I can regulate impulsive behavior in stressful situations at work.	3.34	0.84	0.47
31	I can honestly admit my faults.	3.28	0.85	0.41
32	I can accept feedback openly.	3.28	0.79	0.38
33	I can work in collaboration between team members.	3.30	0.82	0.30
34	I can work in collaboration with an interdisciplinary team.	3.31	0.81	0.30
35	I can maintain a good interpersonal relationship with patients and medical staff.	3.29	0.89	0.33

### Content validity of the NPRS-A

The findings of the evaluation revealed I-CVIs of 1 and S-CVI/Ave of 1. These findings provide evidence of the NPRS-A’s good content validity.

### Construct validity of the NPRS-A

The calculated value of the KMO was 0.92, and the “Bartlett’s Test of Sphericity” was significant (*p* < .001). These values support the conduct of PCA. The PCA determined five distinct components present in the NPRS-A. These components were extracted based on Eigenvalue >1 and factor loadings ≥ 0.40. The "cumulative variance explained" of the five components was 69.2%. Twenty-one items loaded on Component 1 with contributed variance explained of 42.9%. Eight items were loaded in Component 2 (explained variance = 10.8%), while five were loaded in Component 3 (explained variance = 8.3%). Components 4 and 5 have three items, contributing 3.9% and 3.3% of the explained variance, respectively. However, five items (17, 18, 19, 21, and 22) were loaded in Components 1 and 2. After thoroughly examining the items, we decided to retain these items in Component 2. Therefore, the final scale structure is composed of the following: Component 1, "Clinical judgment and nursing performance," with 16 items; component 2, "Professional attitudes," with eight items; component 3, "Patient-centeredness," with five items; Component 4, "Collaborative interpersonal relationship," with three items; and Component 5, "Self-regulation," with three items (Table 3).

**Table 3:** Result of the principal components analysis (n = 373).

Item	Component 1	Component 2	Component 3	Component 4	Component 5
	Clinical judgment and nursing performance	Professional attitudes	Patient-centeredness	Collaborative interpersonal relationship	Self-regulation
Q2	0.834				
Q1	0.812				
Q6	0.790				
Q14	0.790				
Q12	0.777				
Q11	0.763				
Q8	0.750				
Q13	0.747				
Q15	0.739				
Q7	0.728				
Q4	0.724				
Q9	0.703				
Q10	0.700				
Q5	0.686				
Q3	0.662				
Q16	0.649				
Q24		0.758			
Q20		0.757			
Q18	0.412	0.729			
Q21	0.413	0.706			
Q19	0.427	0.703			
Q22	0.438	0.687			
Q23		0.682			
Q17	0.463	0.653			
Q26			0.845		
Q25			0.836		
Q29			0.690		
Q27			0.672		
Q28			0.651		
Q33				0.928	
Q34				0.915	
Q35				0.898	
Q32					0.767
Q30					0.758
Q31					0.708
Eigenvalue	15.01	3.80	2.91	1.35	1.15
Variance explained (%)	42.9	10.8	8.3	3.9	3.3
Cumulative variance explained (%)	42.9	53.7	62.0	65.9	69.2

The test of difference in the nursing practice preparedness between students from different year levels showed statistically significant results (*F* = 55.32, *p* < .001). The Tukey HSD test reflected that students in the 2^nd^ year were lesser prepared to practice nursing as compared to junior (*p* < .001) and senior (*p* < .001) nursing students. The correlation test showed positive correlations, indicating that older students with higher GPAs and a higher level of self-reported readiness to practice nursing are more ready to practice nursing in clinical settings (Table 4).

**Table 4:** Results of the test of construct validity by hypothesis testing method (n = 373).

Respondent’s characteristics	Mean	SD	Statistical test	*p*
Gender				
Male	3.17	0.54	*t* = -0.25	.802
Female	3.19	0.53		
Academic year level^a^				
2nd year	2.79	0.64	*F* = 55.32	< .001***
3rd year	3.31	0.40		
4th year	3.44	0.35		
Age			*r* = 0.29	< .001***
Latest GPA			*r* = 0.18	.001**
Perceived readiness to practice nursing			*r* = 0.38	< .001***

Note. ^a^ 2^nd^ year versus 3^rd^ year (*p* < .001), 2^nd^ year versus 4^th^ year (*p* < .001)

**Significant at .01 level

***Significant at .001 level

### Reliability of the NPRS-A

The analysis showed an overall Cronbach’s alpha of 0.957. For the five components, the Cronbach’s alpha was the following: 0.961 for “Clinical judgment and nursing performance,” 0.946 for “Professional attitudes,” 0.839 for “Patient-centeredness,” 0.949 for “Collaborative interpersonal relationship,” and 0.785 for “Self-regulation” (Table 5).

**Table 5:** Internal consistency reliability of the Nursing Practice Readiness Scale Arabic version (n = 373).

Scale	Cronbach’s alpha
Entire scale	0.957
Component 1: Clinical judgment and nursing performance	0.961
Component 2: Professional attitudes	0.946
Component 3: Patient-centeredness	0.839
Component 4: Collaborative interpersonal relationship	0.949
Component 5: Self-regulation	0.785

## Discussion

**`** A psychometrically sound tool to assess Saudi Nursing Practice Readiness perspective is essential. Thus, this investigation was conducted to test the NPRS-A’s psychometric properties within the framework of Saudi nursing students’ readiness to practice nursing in clinical settings.

The panel of nursing professionals evaluated the CVI of the NPRS-A to determine the applicability of all the items of the NPRS-A for Saudi student nurses. The panel evaluation of the tool revealed an I-CVI of 1 and S-CVI/Ave of 1. This finding shows that the content validity of the 35 questions of the NPRS-A is excellent, which makes the tool acceptable and applicable to the culture and nursing practice in Saudi Arabia. This excellent content validity may be associated with adapting international nursing concepts and the practice of nursing school [17]. Also, the original version of the scale was developed using a rigorous process, including conceptual framework development, extensive literature review, and interviews. The original version’s items were also evaluated by content validity, which was found to be at excellent levels [4]. This study is the first attempt to translate the tool to a different language and test the psychometric properties of the translated version. Hence, comparing the results with previous related studies is a challenge. However, studies reporting the content validity of other tools measuring the same construct showed excellent validity. For instance, Kuleyin and Açıl [18] used the same methods and number of experts to test the Turkish version of the “Casey-Fink Readiness for Practice Scale” content validity and reported a 0.94 S-CVI/Ave.

Before conducting the PCA, ITCs were computed to examine how well a specific item of the scale measures the same construct as the other items. The findings revealed that the ITC values were within the acceptable values (ITCs ≥ 0.30) [16]. This implies that each item in the scale adequately measures the construct of “practice readiness” among Saudi nursing students. These findings further indicate that each scale item was consistent with the behavior of all items on the scale; none were dropped, and all 35 items were entered into the PCA [16]. The PCA with Varimax rotation revealed the five distinct components of NPRS-A, which has a “cumulative variance explained” of 69.2%. The five extracted NPRS-A components met the Eigenvalue >1 and factor loadings ≥ 0.40. This analysis was explained by Tabachnick and Fidell [19], who states that a good construct validity has more than 50% variance. Thus, the NPRS-A “cumulative variance explained” of 69.2% shows a decent construct validity. The five components of the NPRS-A were comparable with the original version of the scale [4]. Consequently, the final NPRS-A components were similarly categorized as “Clinical judgment and nursing performance” (16 items), “Professional attitudes” (8 items), “Patient-centeredness” (5 items), “Collaborative interpersonal relationship” (3 items), and “Self-regulation” (3 items). The similarity between the components of the original version and the Arabic versions’ components is traced to the progressive development of nursing education and practice in Saudi Arabia [20]. Nursing leaders are also adopting the international standard to ensure that nurse graduates are well-trained to achieve the 2030 vision of the government, which is to increase the number of employed Saudi nationals in the healthcare system [21]. Previous studies employed a similar methodology to extract the components of Arabic language adaptation of scales and to support their construct validity [22–24]. Moreover, the “Casey-Fink Readiness for Practice Scale,” which is a similar scale, was found using exploratory factor analysis to have four subscales, namely “clinical problem solving, learning techniques, professional identity, and trials and tribulations” [25].

The findings showed that five items (17, 18, 19, 21, and 22) were loaded in Components 1 and 2. After thoroughly examining the items, the researchers decided to retain these items in Component 2 since the factor loading of the items were higher in this component and these items were strongly associated with the construct being measured by component 2. As Nunnally and Bernstein [16] mentioned, items loaded in two or more components should be retained where they are strongly associated. Furthermore, Direkci et al. [26] state that an item should be retained in a component where the loading factor is above the acceptable level.

The most crucial component of the NPRS-A is the “Clinical judgment and nursing performance,” which has an eigenvalue of 15.01. This finding revealed that student nurses’ clinical judgment and performance matter most in the readiness of their clinical practice; this finding is consistent with the original study [4]. This result could also be related to the gap in the theory and practice experience of the nursing student [27]. This component is related to the “Clinical problem-solving subscale” of the “Casey-Fink Readiness for Practice Scale,” which involves the students’ readiness in problem-solving and decision-making skills as applied in clinical practice [18, 25].

Component 2 focuses on "Professional attitudes," and the five items are practices that the nursing student applies in solving, coping, and performing nursing care independently. These practices talk about professional values since it shows the nursing attitude and values in applying moral and ethical reasoning in their clinical practice and judgment [4, 6]. A professional attitude is vital since it helps personal and organizational development. A professional attitude makes the work environment healthier and more friendly, which creates a harmonious relationship [28]. Also, understanding one potential and ability to cope with a situation or stressful event will make the organization productive [29]. Thus, mental health is a critical aspect in preparing nursing students for their clinical practice, especially for male Saudi student nurses who prefer to be mum if they have psychological problems [4, 30].

The third highest component is “Patient-centeredness.” Upon analyzing the items loaded in this component, their central construct is about ensuring patient privacy, emphasizing and helping the patient vent their needs and concerns, and responding to and respecting them [4]. In nursing, patient-centeredness is essential to improve the therapeutic nurse-patient relationship. Readiness to provide “patient-centered” care among nursing students requires professional commitment, which helps student nurses embrace their professional responsibilities and values [31]. This area of practice was not included in a previous scale developed to assess senior nursing students’ readiness to practice nursing [25].

The fourth component is the "Collaborative interpersonal relationship." The items in this component indicate the readiness of the nursing students to communicate and work harmoniously and competently with another health team member to ensure quality patient care [4]. Effective collaboration ensures that patients receive quality and safe nursing care. According to Balay-odao et al. [28] and Lee and Hwang [32], collaboration in the clinical setting prevents medical error and practice. Furthermore, collaboration prevents interpersonal relationship issues in the clinical setting, which is one of the reasons for poor-quality patient care [33]. The findings imply that Saudi student nurses perceived the importance of effective collaboration among medical staff in the clinical setting. Thus, preparing nursing students in this area during their baccalaureate studies is essential to ensure they can work collaboratively with the healthcare team in their future practice.

Lastly, “Self-regulation" has a low eigenvalue, which could be related to male Saudi nurses’ lack of expression of emotions [34]. Also, student nurses have a high-stress level due to fear of committing a medical error in their clinical practice [17]. Also, student nurses have different perceptions regarding clinical practice, and feedback can positively and negatively affect the student. That is why those giving feedback should be cautious and sensitive to students’ feelings [35].

In addition, the test of correlations indicates that older students with higher GPAs and a higher level of self-reported readiness to practice nursing are more ready to practice nursing in clinical settings. This finding could be associated with the level of development of the nursing student. Those older and at a higher level are more likely to develop coping strategies to adapt and adjust to the demand of their clinical practice [17]. Furthermore, those on higher levels had more clinical exposure, which helped them better understand and perceive their clinical practice. Also, students with higher GPAs manifested better academic and clinical performance during clinical exposure. It was noted that students with high GPAs showed more dedication to learning by reading in advance, being more responsible, and putting extra effort into practicing nursing skills. Also, those with higher GPAs have a high level of assertiveness [36, 37].

The scale’s Cronbach’s alphas result revealed acceptable reliability of the NPRS-A, including all five components, since all the values are higher than the 0.70 threshold value. This reliability result is consistent with the reported reliability of the original tool [4]. The result of the study infers the relatedness of the items and the concept measured, which is the nursing practice readiness of student nurses. Similarly, the results show that the component items are interrelated in measuring the component’s concept.

The researchers acknowledge the limitations of this study. The construct validity needs to be supported by other methods, such as convergent and divergent validity and confirmatory factor analysis. Future studies should consider performing these validity tests to support the psychometric properties of the Arabic version of the scale. The tool’s reliability should also be supported by other methods, such as the test-retest reliability, to establish the stability reliability of the tool. Also, while the sample size was adequate for the study, the representation of 4^th^-year students was the lowest. Future studies should replicate the study to involve larger samples from the final year of the nursing program.

## Conclusions

The study tested the NPRS-A’s psychometric properties when assessing Saudi nursing students’ readiness to practice nursing in clinical settings. Considering the central role of nursing education in preparing nursing students to practice nursing in the real world, ensuring that nursing students are prepared to practice nursing is critical. Regular assessments of the student’s readiness to practice nursing are necessary to identify the student’s weak points and implement necessary interventions to improve these weaknesses. This critical part of nursing education entails a valid and reliable tool to accurately assess students’ readiness. The study supported the NPRS-A’s content and construct validity, and the internal consistency reliability. The study concludes that the NPRS-A is a valid and reliable tool that can accurately measure Saudi nursing students’ readiness to practice nursing. The establishment of the NPRS-A is significant, not only in Saudi Arabia but other Arabic-speaking countries. Nurse educators and nursing education policymakers can use this version to assess nursing students’ learning needs to be prepared to practice nursing. The tool can also be used to plan, implement, and evaluate educational (theoretical and practical) interventions to ensure the nursing students’ readiness to practice in the clinical setting. Clinical preceptors can also use the tool to assess the readiness of their preceptee to practice clinical nursing. Lastly, the tool opens new opportunities for research in this area of nursing education, as well as cross-cultural studies that can compare the readiness to practice nursing among students from various parts of the world.

## Supporting information

S1 Checklist STROBE statement. Checklist of items that should be included in reports of cross-sectional studies.
(DOCX)
